# Deep medullary veins score is associated with atrophy in patients with cerebral small vessel disease

**DOI:** 10.3389/fneur.2024.1417805

**Published:** 2024-08-29

**Authors:** Fan Mao, Zhihua Xu, Meihua Shao, Xuelian Xiang, Xiaoli Zhou

**Affiliations:** Department of Radiology, Tongde Hospital of Zhejiang Province, Hangzhou, China

**Keywords:** deep medullary vein, cerebral small vessel disease, atrophy, susceptibility weighted imaging, age

## Abstract

**Objective:**

To explore the relationship between the deep medullary vein (DMV) score and atrophy in patients with cerebral small vessel disease (CSVD).

**Methods:**

Imaging and clinical data from 125 patients with CSVD from January to December 2022 were reviewed. Normalized gray matter volume (GM_N) was calculated by dividing the gray matter volume by the whole brain volume. DMV scoring is conducted using susceptibility-weighted magnetic resonance imaging, wherein the DMV area is partitioned into six distinct regions: bilateral frontal, parietal, and occipital regions. Each region undergoes assessment based on the clarity and consistency of DMV visibility. Subsequently, the scores from these six regions are summed, resulting in a score ranging from 0 to 18 points.

**Results:**

DMV score was associated with GM_N (*r* = −0.376, *p* < 0.001). Comparisons among patients according to GM_N tertiles, differences in gender, age, current smoking, DMV score, and total CSVD magnetic resonance imaging score were demonstrated (*p* < 0.05). Adjusting for age, gender, vascular risk factors, and total CSVD MR score, the DMV score was independently associated with GM_N [*β* (95% CI): −0.347 (−0.525, −0.168), *p* < 0.001].

**Conclusion:**

DMV scores are independently associated with GM_N, and DMV dysfunction may play a role in brain atrophy.

## 1 Introduction

Cerebral small vessel disease (CSVD) is a contributor to stroke, vascular cognitive impairment and dementia, primarily affecting the elderly population. It encompasses a range of abnormalities impacting the small arteries, arterioles, capillaries, and venules in the brain. Common imaging markers of CSVD include white matter hyperintensities, lacunar infarcts, cerebral microbleeds, perivascular space and brain atrophy (1). Atrophy, in particular, is also observed in the neurodegenerative processes of other neurological disorders such as Alzheimer’s disease (AD). It is often associated with cognitive impairment (2), significantly impacting their quality of life. However, the underlying mechanism of atrophy remains elusive.

In recent years, the widespread adoption of susceptibility-weighted magnetic resonance imaging (SWI) has brought attention to the investigation of brain venous disruption in the pathophysiology of CSVD among clinicians and researchers (3, 4). SWI is a highly sensitive technique that exploits magnetic susceptibility differences between tissues to provide detailed images of venous structures and iron deposition in the brain. This imaging modality has been instrumental in enhancing our understanding of various neurological conditions, including CSVD. Current research indicates that the assessment of the deep medullary veins (DMV) on SWI reflects the degree of their visibility (5), serving as an indicator of luminal narrowing (6). The DMVs are crucial for draining blood from the deep white matter regions of the brain. Luminal narrowing or dysfunction in these veins can lead to impaired blood flow and subsequent damage to the surrounding brain tissue. Numerous studies have also identified correlations between DMV scores and CSVD imaging markers or overall disease burden (7, 8).

Furthermore, emerging evidence suggests that DMV dysfunction contributes to the onset and progression of CSVD by augmenting interstitial fluid content within the brain’s white matter (9), potentially leading to CSVD-related cognitive decline (10). The accumulation of interstitial fluid can disrupt the microstructural integrity of white matter tracts, impairing neuronal communication and cognitive function. This mechanism highlights the importance of venous health in maintaining brain homeostasis and preventing cognitive deterioration.

Nevertheless, the relationship between DMV dysfunction and atrophy remains ambiguous. Consequently, this study aims to elucidate the association between DMV scores and gray matter volume in patients with CSVD through the use of multimodal imaging techniques.

## 2 Materials and methods

### 2.1 Participants

**Figure 1:**
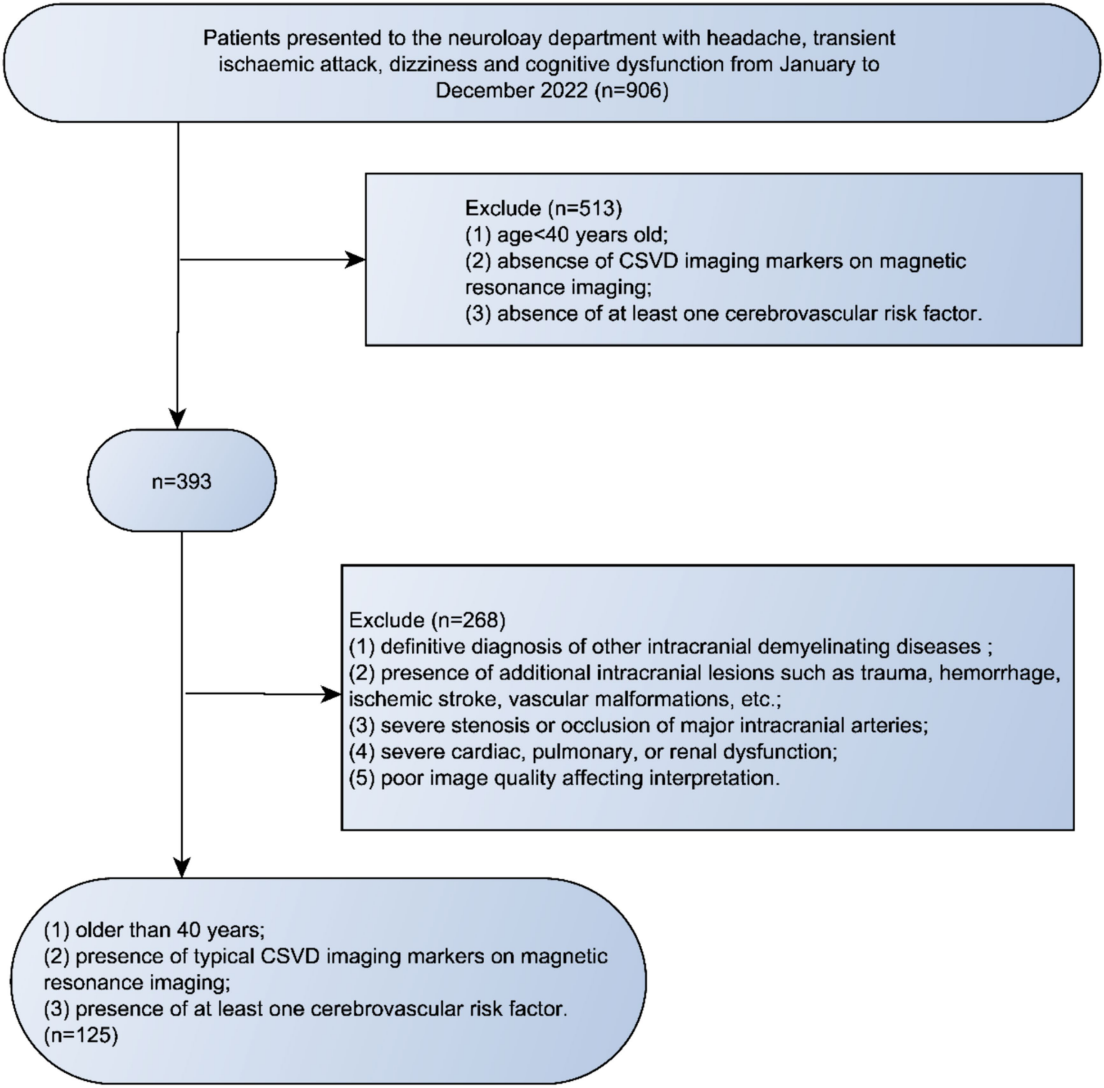
Flowchart of the enrollment of study patients. CSVD, cerebral small vessel disease.

This study protocol underwent review by the Ethics Committee of our institution, and all participants provided informed consent. We conducted an analysis of the imaging and clinical data from patients with CSVD spanning January to December 2022. Inclusion criteria comprised: (1) older than 40 years; (2) presence of typical CSVD imaging markers on magnetic resonance imaging; and (3) presence of at least one cerebrovascular risk factor. Exclusion criteria included: (1) definitive diagnosis of other intracranial demyelinating diseases such as inflammatory or genetic conditions; (2) presence of additional intracranial lesions such as trauma, hemorrhage, ischemic stroke, vascular malformations, etc.; (3) severe stenosis or occlusion of major intracranial arteries; (4) severe cardiac, pulmonary, or renal dysfunction; and (5) poor image quality affecting interpretation. A flowchart of the enrollment of study patients was shown in Figure 1.

### 2.2 MRI protocol

All patients underwent comprehensive magnetic resonance imaging examinations, encompassing standard sequences such as T1-weighted imaging, T2-weighted imaging, and T2 fluid attenuated inversion recovery, as well as advanced techniques including 3D T1-weighted imaging (3D T1WI), time-of-flight magnetic resonance angiography (TOF-MRA), and SWI. The pertinent parameters are delineated below: (1) 3D T1WI: repetition time (TR) = 2000 ms, echo time (TE) = 2.84 ms, slice thickness = 1 mm, matrix size = 256 × 256. (2) SWI: TR = 27 ms, TE = 20 ms, flip angle = 10°, slice thickness = 2 mm, and matrix size = 512 × 512.

### 2.3 Image analyses

#### 2.3.1 Normalization of gray matter volume

To normalize gray matter volume, we first convert the 3D T1-weighted DICOM images into the Neuroimaging Informatics Technology Initiative (NIfTI) format. This conversion is essential for ensuring compatibility with subsequent neuroimaging analysis tools. We then employ the fsl_anat pipeline for comprehensive brain segmentation. This process includes the segmentation of brain gray matter and the creation of a whole brain mask. Using these segmentations, we quantify the volumes of both brain gray matter and the entire brain. Finally, we compute the normalized gray matter volume (GM_N) by dividing the gray matter volume by the whole brain volume. This normalization step is crucial for accounting for individual differences in brain size, allowing for more accurate comparisons across subjects.

#### 2.3.2 DMV score

**Figure 2:**
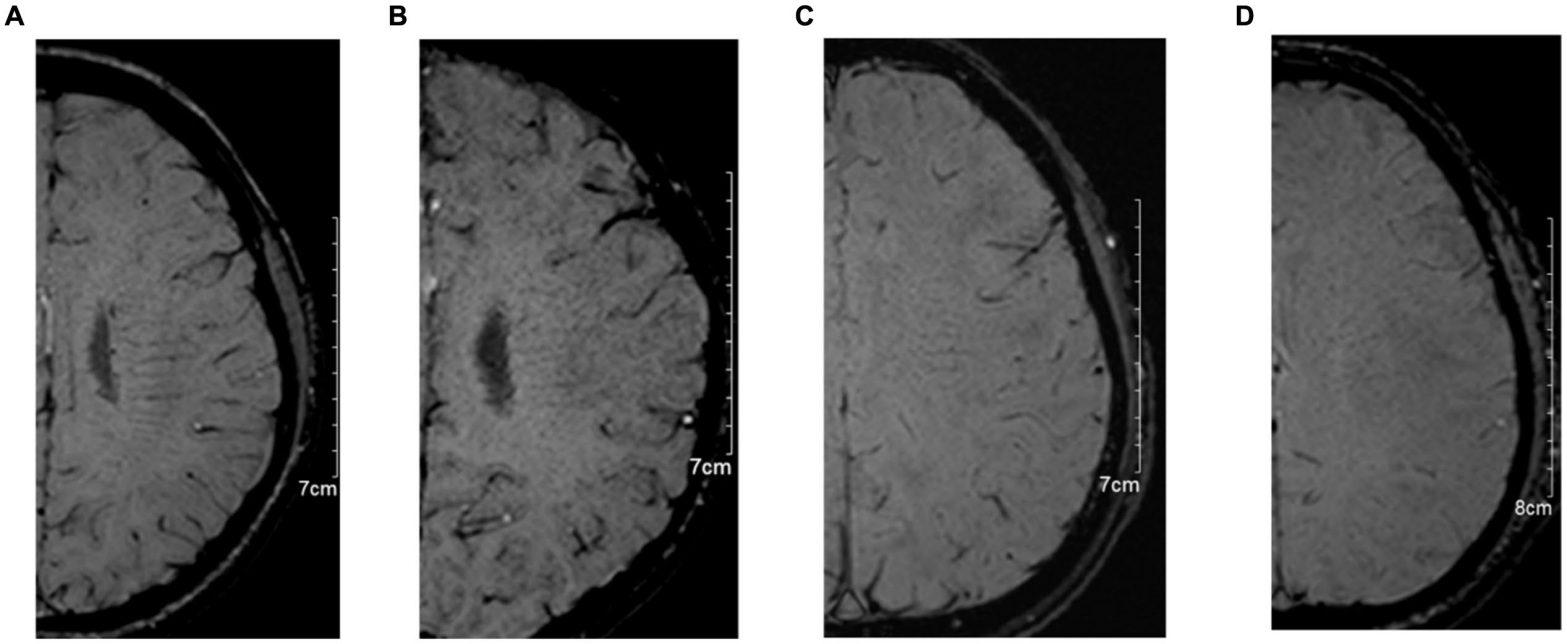
The typical images for deep medullary vein (DMV) scoring system. **(A)**: 0 points for pronounced DMV visibility with consistent signal; **(B)**: 1 point for relatively clear DMV visibility, notwithstanding at least one instance of signal interruption; **(C)**: 2 points for somewhat blurred DMV visibility with signal disruption; and **(D)**: 3 points for indistinct DMV visibility.

In the evaluation of DMV, SWI images are utilized, wherein the DMV region is delineated into six specific areas encompassing the bilateral frontal, parietal, and occipital lobes according to anatomy (11). Each area undergoes assessment based on the clarity and consistency of DMV visibility on SWI images (5). Subsequently, scores from these six regions are aggregated, yielding a total score ranging from 0 to 18 points. The DMV scoring system was used as a series of previous studies (4, 5, 8). A score of 0 denotes robust DMV visibility with uninterrupted signal, whereas a score of 18 signifies the absence of DMV visibility. The scoring criteria for DMV are as follows: 0 points for pronounced DMV visibility with consistent signal; 1 point for relatively clear DMV visibility, notwithstanding at least one instance of signal interruption; 2 points for somewhat blurred DMV visibility with signal disruption; and 3 points for indistinct DMV visibility (see Figure 2). The DMV score was independently assessed by two experienced neuroradiologists (FM and ZX), each with over 5 years of experience in neuroimaging. To ensure unbiased scoring, both readers were blinded to the clinical data and other imaging results.

#### 2.3.3 Total CSVD MR score

The total CSVD MR score is determined by the presence of high-grade white matter hyperintensities, microbleeds, high-grade basal ganglia perivascular spaces enlargement, and lacunar infarcts (12). Each presence contributes 1 point, with a maximum score of 4 and a minimum score of 0. High-grade white matter hyperintensities are defined as Fazekas scores of 2 or higher adjacent to the lateral ventricles or within deep white matter. High-grade perivascular spaces enlargement is defined as unilateral basal ganglia perivascular spaces enlargement exceeding a count of 10. The total CSVD MR score was independently assessed by two additional neuroradiologists (MS and XX), each with over 5 years of experience.

### 2.4 Statistical analyses

For normally distributed quantitative data, mean ± standard deviation is used, while for non-normally distributed quantitative data, quartiles are employed. Qualitative data are presented as frequencies. Participants are divided into three groups based on the tertiles of GM_N, and univariate analysis is conducted to identify factors associated with GM_N. Multiple factor analysis of the correlation between DMV scores and GM_N is performed using linear regression analysis. Statistical analysis is conducted using SPSS 20.0 software, with statistical significance set at *p* < 0.05.

## 3 Results

In total, 125 patients (57 males and mean age: 60 ± 11 years) with CSVD were enrolled in this study. Of them, there were 67 (53.6%) with hypertension, 22 (17.6%) with diabetes mellitus, 27 (21.6%) with current smoking, and 31 (24.8%) with hyperlipidemia. The median (IQR) DMV score, total CSVD MR score, and GM_N were 3 (1, 7), 1 (0, 2), and 0.23 (0.22, 0.24), respectively.

### 3.1 Inter-reader agreement in evaluation of DMV score and total CSVD MR score

All readers were blinded to the clinical data and other imaging results to ensure unbiased scoring. The inter-reader intraclass correlation coefficients (ICCs) for the DMV score on SWI and the total CSVD MR score were 0.92 and 0.99, respectively, indicating excellent agreement between the readers.

### 3.2 Univariate analysis for factors associated with GM_N

**Figure 3:**
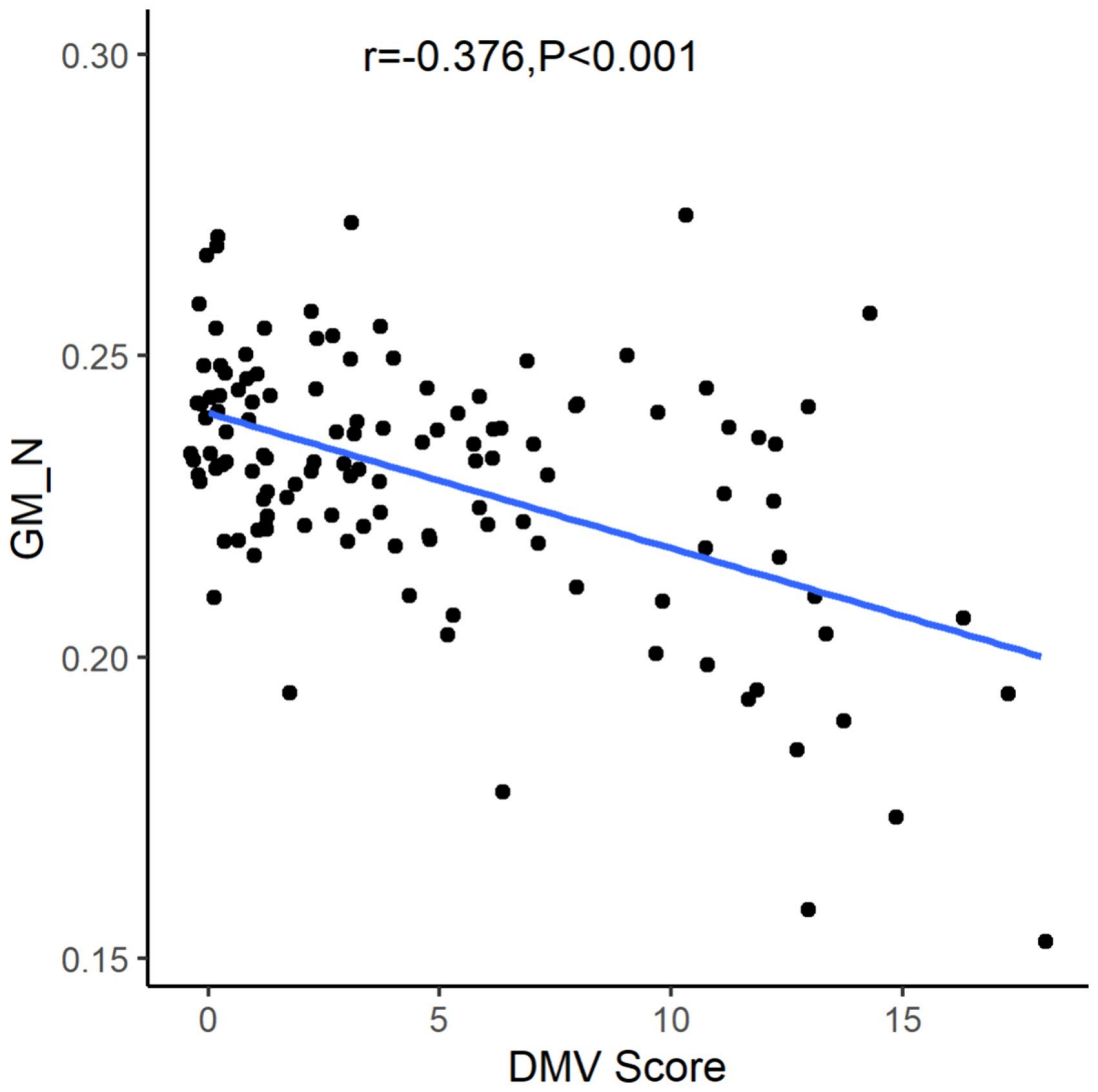
The relationship between gray matter volume and DMV score. DMV, deep medullary vein; GM_N, normalized gray matter volume.

After the Spearman correlation coefficient, the DMV score was associated with GM_N (*r* = −0.376, *p* < 0.001, see Figure 3). Further, comparisons among patients according to GM_N tertiles, the differences in gender, age, current smoking, DMV score, and total CSVD MR score are shown (*p* < 0.05, see Table 1). However, no significant correlations were found between GM_N and hypertension, diabetes mellitus, or hyperlipidemia (*p* > 0.05, see Table 1).

**Table 1:** Clinical and imaging characteristics among participants according to gray matter volume tertiles.

	GM_N tertiles			*p*-value
*n*	42	42	41	
Male	26 (61.9)	15 (35.7)	16 (39.0)	0.032
Age	66 ± 10	55 ± 9	57 ± 11	<0.001
Hypertension	26 (61.9)	17 (40.5)	24 (58.5)	0.107
Diabetes mellitus	11 (26.2)	4 (9.5)	7 (17.1)	0.133
Current smoking	15 (35.7)	7 (16.7)	5 (12.2)	0.021
Hyperlipidemia	10 (23.8)	11 (26.2)	10 (24.4)	0.966
DMV score	6 (3, 12)	3 (1, 6)	1 (0, 5)	<0.001
Total CSVD MR score	2 (0, 3)	0 (0, 1)	0 (0, 1)	0.002
GM_N	0.21 (0.20, 0.22)	0.23 (0.23, 0.24)	0.25 (0.24, 0.25)	<0.001

DMV, deep medullary vein; CSVD, cerebral small vessel disease. GM_N, normalized gray matter volume.

### 3.3 Multivariable analysis for factors associated with GM_N

After a multivariable logistic analysis adjusting for age, gender, vascular risk factors, and total CSVD MR score, the DMV score was independently associated with GM_N [*β* (95% CI): −0.347 (−0.525, −0.168), *p* < 0.001, see Table 2].

**Table 2:** Multivariate analysis for factors associated with gray matter volume.

	*β* value	*p*-value	95.0% confidence interval for *β*	
			Lower bound	Upper bound
Male	−0.131	0.149	−0.310	0.048
Age	−0.175	0.098	−0.383	0.033
Hypertension	0.138	0.106	−0.030	0.305
Diabetes mellitus	−0.085	0.282	−0.240	0.071
Current smoking	−0.059	0.516	−0.239	0.121
Hyperlipidemia	−0.012	0.881	−0.167	0.144
DMV score	−0.347	0.000	−0.525	−0.168
Total CSVD MR score	−0.150	0.132	−0.345	0.046

DMV, deep medullary vein; CSVD, cerebral small vessel disease.

## 4 Discussion

The results of this study indicate that higher DMV scores are associated with lower GM_N values, suggesting that more prominent DMV dysfunction is correlated with a smaller gray matter volume and more evident brain atrophy. Furthermore, after adjusting for age, gender, vascular risk factors, and total CSVD MR score, DMV scores remain independently associated with GM_N. Therefore, we hypothesize that DMV dysfunction may play an important role in brain atrophy.

In patients with CSVD, the decreased visibility of DMV on SWI images may be primarily attributed to: (1) chronic hypoxic and hypometabolic state of brain tissue in patients with CSVD (13); and (2) deposition of collagen proteins in the DMV vessel walls, leading to luminal narrowing (6).

SWI imaging is known for its sensitivity to blood oxygenation levels, with low-oxygenated blood potentially appearing less prominent in the images. Consequently, in conditions such as hypoxia or abnormal blood oxygen levels, the visualization of veins might be diminished. As compared with that of the other intracranial veins, DMV may reflect deoxygenated hemoglobin levels and brain metabolism more sensitively (14). Studies by Yin et al. (15) and Chen et al. (7) have established correlations between DMV scores and markers of CSVD imaging markers, as well as CSVD burden. Building upon this research, our study highlights a significant correlation between DMV scores and GM_N. Consequently, we hypothesize that alterations in DMV function may impact brain structural changes extensively and globally, rather than solely locally. This hypothesis aligns with current research emphasizing CSVD-related global hypoperfusion and hypometabolism (16).

From another perspective, the accumulation of collagen proteins within the vessel walls of the DMV vessel walls can lead to luminal narrowing and increased venous pressure, resulting in interstitial edema in the brain white matter (9, 17). A study by Man et al. (17) found increased free water content in the normal-appearing white matter of patients with CSVD. Elevated interstitial fluid can lead to the deposition of harmful substances in brain tissue (18), potentially causing damage to white matter microstructures (e.g., myelin and axons). Damage to white matter fiber bundles may lead to neuronal malnutrition, manifesting as brain atrophy. Additionally, DMV may not only drain white matter but also gray matter. Dysfunction of DMV may directly impact gray matter when drainage is impaired. Certainly, the detailed mechanism warrants further investigation and exploration.

The correlation coefficient of −0.376, while statistically significant, indicates a weak correlation between DMV score and GM_N. This suggests that while there is an association between DMV scores and GM_N, other factors not accounted for in this study may also play significant roles in determining gray matter volume. This weak correlation could reflect the complex and multifactorial nature of brain atrophy in CSVD, such as damage of blood-brain barrier (19–21), neuroinflammation, where DMV dysfunction is only one of several contributing factors. Moreover, venous disruptions may promote blood–brain barrier disruption and neuroinflammation forming a vicious cycle (22), which need a further study.

This study also has certain limitations. First, it is a single-center study with a relatively small sample size. Second, the DMV scoring adopted in this study is semi-quantitative. Perhaps using artificial intelligence for automated segmentation could offer a better interpretation of DMV changes. Finally, there was no follow-up conducted to observe the relationship dynamically between changes in DMV and brain atrophy. To address the abovementioned limitations, more multicenter investigations with more patients are required.

In conclusion, we found that DMV scores are independently associated with GM_N, and DMV dysfunction may play a role in brain atrophy. DMV dysfunction may lead to widespread changes throughout the brain, warranting further investigation in the future.

## Data Availability

The original contributions presented in the study are included in the article/supplementary material, further inquiries can be directed to the corresponding author.
